# Sex and gender determinants following spinal fusion surgery: A systematic review of clinical data

**DOI:** 10.3389/fsurg.2022.983931

**Published:** 2022-10-17

**Authors:** Francesca Salamanna, Deyanira Contartese, Matilde Tschon, Veronica Borsari, Cristiana Griffoni, Alessandro Gasbarrini, Milena Fini

**Affiliations:** ^1^ Complex Structure Surgical Sciences and Technologies, IRCCS Istituto Ortopedico Rizzoli, Bologna, Italy; ^2^ Spine Surgery, IRCCS Istituto Ortopedico Rizzoli, Bologna, Italy; ^3^ Scientific Direction, IRCCS Istituto Ortopedico Rizzoli, Bologna, Italy

**Keywords:** spinal fusion surgery, clinical data, systematic review, sex, gender differences

## Abstract

In the last decade, numerous studies analyzed and described the surgical outcomes in male and female patients submitted to orthopedic surgery. Although this, the impact of sex/gender on spinal fusion surgery clinical outcomes is still poorly defined. This review systematically maps and synthesizes the scientific literature on sex/gender differences in postoperative outcomes for patients undergoing spinal fusion surgery. The search was performed in PubMed, Scopus, and Web of Science in the last 22 years. Clinical studies evaluating potential sex/gender differences in postoperative outcomes and/or complications, as primary or secondary aim, were included and analyzed. Out of the 1,885 records screened, 47 studies were included. These studies comprised a total of 1,158,555 patients (51.31% female; 48.69% male). About 77% of the analyzed studies reported sex/gender-related differences in postoperative outcomes. Most studies treated patients for lumbar degenerative diseases and more than 55% of them reported a worse postoperative outcome in female patients in terms of pain, disability, health-related quality of life questionnaires, and complications. Differently, a significant heterogeneity across studies on patients treated for cervical and sacral degenerative diseases as well as for spinal deformity and traumatic spinal fracture prevented the understanding of specific sex/gender differences after spinal fusion surgery. Despite this, the present review highlighted those female patients treated for lumbar degenerative spine diseases could require more clinical awareness during postoperative care. The understanding of how sex/gender differences can really affect clinical outcomes after spinal fusion surgeries is mandatory for all spinal pathological conditions to drive clinical research toward oriented and personalized protocols.

## Introduction

In the last decade, sex/gender differences attracted considerable interest in several specialties, including cardiothoracic and trauma surgery (1). These differences are key issues for a personalized treatment. The National Institutes of Health (NIH) promotes investigators to design their studies in a way that allows the participants to self-identify their sex or gender (2). By sex, we mean the biological character of male and female as opposed to gender, which reflects societal roles and expectations (2). Although, sex and gender are two different concepts, in medicine they are often linked, and, in the review, they were used as synonyms. Within orthopedic surgery, a patient's sex/gender is thought to influence outcomes after total joint arthroplasty (manly in total knee arthroplasty), after rotator cuff repair, and after anterior shoulder surgical stabilization (3). Sex-based differences in spinal disease incidence have been previously described in several studies, such as lumbar degenerative disc disease (4), lumbar radiculopathy (5), and cauda equina syndrome (6). Nevertheless, Taylor et al. (7) highlighted that male patients generally receive more recommendations than female ones for spine surgery despite similar underlying disorders (7). The impact of male or female sex/gender on clinical outcomes in spinal fusion surgery is less well defined. This is in part because most of the studies do not consider patient sex when examining demographic trends in outcomes, reporting “sex-adjusted” statistics, or using sex-matched groups. Despite this, some data demonstrated individual differences in the postoperative outcomes between male and female patients after spinal fusion surgery. In detail, several clinical trials found a better global and functional outcome after lumbar spine fusion for males than for females. A retrospective study by Gehrchen et al. (8) reported that the female gender is an independent risk factor for nonoptimal outcomes after lumbar fusion; a randomized controlled trial (9) observed that the female gender was also associated with worse postoperative results (10). However, a prospective clinical study on 4,780 patients in the Swedish National Spine Register with lumbar degenerative disc disease and chronic low back pain showed that female patients had worse pain and function preoperatively but improved more than males after surgery (11). These conflicting results are due to the fact that several of the studies are limited to small samples size, which has insufficient power to reach accurate conclusions. Regarding complications, there are several hypothetical reasons for male and female differences after spinal surgery. Differences in anthropomorphic parameters, body mass index, and comorbidity may contribute to differences in complications and mortality risks between females and males after spine surgery (12–14). Additionally, specific differences in tobacco and alcohol use/abuse as well as in unhealthy lifestyles (malnutrition, unhealthy diet, drug abuse, etc.) might also increase the odds of postoperative death or the development of specific complications, such as surgical site infection (SSI) (15).

The number of studies suggesting that sex/gender may affect patient outcomes after a spinal fusion procedure begets the need for a comprehensive examination of these data. Thus, we performed a systematic review of the literature to better understand sex/gender-based differences in spinal fusion surgery outcomes. These aspects are of key interest for both physician and patient thus, to be informed about predictive gender and sex-related differences. This information could also have a prognostic value, playing a critical role in the decision-making process.

## Methods

### Eligibility criteria

The PICOS model (Population, Intervention, Comparison, Outcomes, Study design) was used to design this study: (1) studies that considered female and male patients (Population) submitted to, (2) spinal fusion surgery (Interventions), (3) with a comparison between them (female and male) (Comparisons), (4) that reported postoperative clinical and/or functional outcomes of spinal fusion surgery (Outcomes), in (5) randomized, retrospective, prospective and case series studies (Study design). Studies from May 2000 to May 2022 were included in this review if they met the PICOS criteria. We excluded studies that evaluated (1) surgeries other than the spine, (2) patients undergoing spine surgery with other severe pathological conditions (genetic and rare diseases, diffuse metastases, advanced neurodegenerative disorders), and (3) articles with incomplete outcomes or data. Additionally, we excluded reviews, case reports, letters, comments to editors, *in vivo* and *in vitro* studies, pilot studies, meta-analyses, editorials, protocols and recommendations, guidelines, and articles not written in English.

### Search strategies

Our literature review involved a systematic search conducted in May 2022. We performed our review according to the Preferred Reporting Items for Systematic Reviews and Meta-Analyses (PRISMA) statement (16). The search was carried out on three databases: PubMed, Scopus, and Web of Science Core Collection. The following combination of terms was used (spinal fusion OR spinal arthrodesis OR vertebral fusion OR vertebral arthrodesis) AND (gender differences OR sex differences OR gender-specific OR sex-specific), and for each of these terms, free words, and controlled vocabulary specific to each bibliographic database were combined using the operator “OR.” The combination of free-vocabulary and/or Medical Subject Headings (MeSH) terms for the identification of studies in PubMed, Scopus, and Web of Science Core Collection were reported in Supplementary Table S1.

### Selection process

After submitting the articles to a public reference manager (Mendeley Desktop 1.19.8) to eliminate duplicates, possible relevant articles were screened using the title and abstract by three reviewers (FS, DC, and MT). Studies that did not meet the inclusion criteria were excluded from review and any disagreement was resolved through discussion until a consensus was reached, or with the involvement of a fourth reviewer (MF). Subsequently, the remaining studies were included in the final stage of data extraction.

### Data collection process and synthesis methods

The data extraction and synthesis process started with cataloging the studies' details. To increase validity and avoid omitting potentially findings for the synthesis, three authors (MT, VB, and CG) extracted and performed tables taking into consideration: the study design, female and male patients' number and age, type of surgery (indication and operation types), presence of comorbidities, complications, main objectives related to sex/gender, follow-up, assessment measures, pre-and postoperative quantitative measures, complications, and outcomes/endpoints (specific sex/gender differences).

### Assessment of methodological quality

The methodological quality of the selected studies was independently assessed by two reviewers (DC and FS), using the Cochrane risk-of-bias tool RoB for randomized trials and the Cochrane risk of bias ROBINS-I for nonrandomized studies of interventions (17). The tool for randomized trials included five domains, which assessed the possible sources of bias: bias arising from the randomization process, bias due to deviations from intended interventions, bias due to missing outcome data, bias in the measurement of the outcome, and bias in the selection of the reported result. For each domain were assigned one of three levels: low risk of bias, some concerns, or high risk of bias until an overall bias risk judgment is reached. The tool for nonrandomized trials included seven domains, which assessed the possible sources of bias: bias due to confounding, bias in the selection of participants into the study, bias in classification of interventions, bias due to deviations from intended interventions, bias due to missing data, bias in the measurement of the outcome, and bias in the selection of the reported result. For each domain were assigned one of three levels: low risk of bias, moderate risk of bias, or high risk of bias until an overall bias risk judgment is reached. In case of disagreement, the reviewers attempted to reach a consensus by discussion; if this failed, a third reviewer (MF) was consulted to make the final decision.

## Results

### Study selection and characteristics

**Figure 1:**
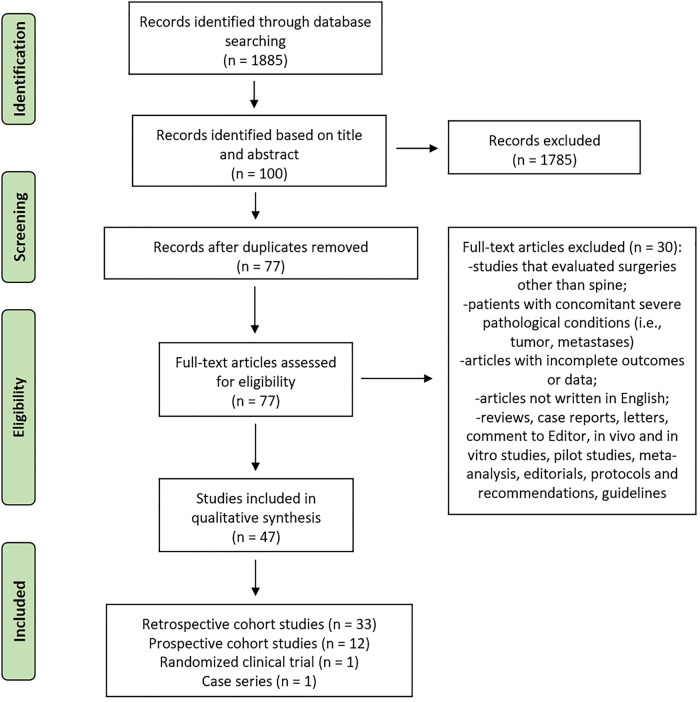
Systematic review flow diagram. The PRISMA flow diagram for the systematic review detailing the database searches, the number of abstracts screened, and the full texts retrieved.

The initial literature search retrieved 1,885 studies. Of those, 798 studies were identified using PubMed, 435 using Scopus, and 652 were found in the Web of Science Core Collection. Articles were screened for title and abstract and 100 articles were selected. Subsequently, these articles were submitted to a public reference manager to eliminate duplicates. The resulting 77 complete articles were then reviewed to establish whether the publications met the inclusion criteria, and 47 studies were considered eligible for this review. Search strategy and study inclusion and exclusion criteria are detailed in Figure 1. Of these articles, 33 were retrospective studies, 12 were prospective studies, 1 was a case series, and another 1 was a randomized clinical trial (RCT).

### Assessment of methodological quality

In our quality assessment, the randomized trial is judged to raise some concerns, but not to be at high risk of bias for any domain. The risk of bias was mainly about the randomization process and measurement of the outcome. The 70% of the nonrandomized studies were rated at low risk of bias for all domains, 21% were rated at moderate risk of bias, and 9% were rated at critical risk of bias. Methodological weaknesses that led to moderate or weak quality scores often included bias due to confounding, missing data, deviations from intended interventions, and bias in the measurement of the outcome. Risks of bias assessments for each randomized and nonrandomized study were reported in Supplementary Tables S2 and S3.

### Studies general information, objectives, and cohort characteristics

Descriptive characteristics of each study are presented in Tables 1 and 2. The included studies involved a total of 1,158,555 patients. The overall percentages of female and male patients were 51.31% and 48.69%, respectively. Apart from five studies evaluating adolescent patients, the others consider patients operated at a mean age of 55.55 ± 7.38 years. Most studies (*n* = 23) examined the association between sex/gender and clinical outcomes in relation to a general clinical assessment and/or to specific scores. Eighteen studies also evaluated the association between sex/gender and postoperative complications, such as intensive care unit (ICU) admission, superficial and deep SSI, postoperative fractures, cardiac events, heterotopic ossifications, instrumentation failure and reoperation rate, length of stay (LOS), or specific risk factors (opioid use, smoking, obesity). Furthermore, one study examined sex/gender differences in relation to recombinant human bone morphogenetic protein-2 (rhBMP2) use during spinal fusion surgery.

**Table 1:** Studies general information.

Reference	Country of publication	Study design	Patients number	Sex/Age	Diagnoses and surgery	Comorbidities	Main objectives related to sex/gender
Abousamra et al. (18)	United States	Retrospective	837	F = 697 M = 140 Mean age: 15.2 ± 2.2	AIS Posterior fusion	NR	Blood loss
Adogwa et al. (19)	United States	Retrospective	13,257	F = 7,871 M = 5,386 Age > 25	Degenerative diseases (symptomatic stenosis or spondylolisthesis) Decompression and one, two, or three-level posterior lumbar instrumented fusion	Direct comparison revealed that female cohort had a higher prevalence of obesity, while males had greater proportions of type 2 diabetes mellitus, myocardial infarction, atrial fibrillation	Differences in opioid use
Alomari et al. (20)	United States	Retrospective	21,180	Single-level group: F = 5,026, M = 6,168 Multilevel group: F = 4,953, M = 5,033	Single- and multilevel ACDF	M: myelopathy, diabetes mellitus, hypertension, bleeding disorders	Peri-op outcomes after ACDF
Alomari et al. (21)	United States	Retrospective	44,526	-Single-level PLF: F = 4,705, M = 4,156 -Multilevel PLF: F = 2,563, M = 2,291 -Single-level PLIF/TLIF: F = 7,737, M = 7,403 -Multilevel PLIF/TLIF: F = 3,481, M = 3,372 -Single-level ALIF/LLIF: F = 2,281, M = 2,139 -Multilevel ALIF/LLIF: F = 2,291, M = 2,107	Degenerative diseases Posterior lumbar fusion, posterior/transforaminal lumbar interbody fusion, anterior/lateral lumbar interbody fusion	Females were older and functionally dependent. Male were more likely to have diabetes mellitus, hypertension, and bleeding disorders	Peri-op outcomes after elective lumbar fusion spine surgery
Ayhan et al. (22)	Europe	Retrospective	199	F = 164 M = 35 Mean age: 51.94	Scoliosis, thoracic kyphosis, degenerative or idiopathic deformity	NR	Clinical outcomes
Basques et al. (23)	United States	Retrospective	20,383	F = 10,486 M = 9,897 Age > 18	Degenerative spine disease ACDF	Male slightly older, ↑ incidence of diabetes and hypertension	Differences in baseline characteristics and risk factors for adverse outcomes
Bumpass et al. (24)	United States	Retrospective	409	F = 319 Mean age: 55.0 M = 90 Mean age: 58.3	Adult spinal deformity Primary posterior instrumented fusion ≥5 levels	NR	Complications, operative morbidity, deformity x-ray correction, HRQoL
Buttermann et al. (25)	United States	Prospective	159	101 females 58 males Mean age: 46.4 ± 9.7	c20 of scoliosis or kyphosis ACDF with or without decompression	NR	Clinical outcomes after ACDF
Chan et al. (26)	United States	Retrospective	477	F = 159 M = 318	Grade 1 degenerative lumbar spondylolisthesis	Diabetes, anxiety, coronary artery disease, osteoporosis, depression	Patients’ satisfaction
Chaichuangchok et al. (27)	Asia	Retrospective	54	F = 19 M = 35 Mean age: 58.9 ± 12	Degenerative cervical spondylitis, myelopathy Single- and multilevel ACDF	Diabetic mellitus, hypertension, dyslipidemia	Predictive factors for the outcomes after ACDF
Christian et al. (28)	United States	Retrospective	536	F = 234 M = 302 Age >65	Degenerative diseases Elective spinal surgery (PCDF, ACDF, thoracic or lumbar short segment (≤4), and thoracic or lumbar long segment (≥5) decompression and/or fusion procedures)	NR	Associations between smoking and pre-op opioid consumption and their impact on post-op outcomes
Elsamadicy et al. (29)	United States	Retrospective	4,972	F = 3,282 M = 1,690	Adult spine deformity Elective spine fusion surgery involving ≥4 levels	Comorbidities: alcohol use, hypertension, diabetes mellitus, overweight/obese, hypercoagulable state, heart failure, atrial fibrillation, chronic obstructive pulmonary disease, chronic kidney disease, peripheral vascular disease, liver disease. Male cohort ↑ prevalence of comorbidities than the female cohort	LOS and discharge disposition after elective spine fusion surgery
Gulbrandsen et al. (30)	United States	Retrospective	1,931	F = 1,219 Mean age: 61 ± 12.83 M = 712 males Mean age: 59 ± 14.07	Lumbar degenerative conditions and thoracolumbar deformity Open posterior instrumented fusion	Comorbidities: autoimmune and gastrointestinal disorders, depression, fibromyalgia, thyroid disease Females had a ↑ number of comorbidities compared to males: F:1.75 ± 1.42 M: 1.50 ± 1.33	Recovery after spinal surgery (pain, function, complications)
Helenius et al. (31)	Europe	Retrospective	60	F = 30 Mean age:16.2 ± 2.6 M = 30 Mean age:15.5 ± 2.3	AIS	None	Long-term results of operative treatment
Hermansen et al. (32)	Europe	RCT	72	F = 39 M = 33 Mean age: 59 ± 8.6	Degenerative radiculopathy with or without neck pain Cervical intervertebral fusion cage or Cloward procedure	NR	Pre-op factors predicting good long-term outcomes and subgroup differences at the 10-year follow-up between patients with and without clinically relevant improvement
Heyer et al. (33)	United States	Retrospective	41,315	F = 20,248 Mean age: 57.27 M = 21,067 Mean age: 56.06	Different spine diseases	Diabetes, severe COPD, hypertension, revascularization/amputation for peripheral vascular disease, preoperative transfusion, bleeding disorder, previous percutaneous coronary intervention, cardiac surgery Male: ↑ bleeding disorder, previous percutaneous coronary intervention, cardiac surgery	30-day complications
Jang et al. (34)	East Asia	Retrospective	208	F = 104 M = 104 Mean age: 45.9	Thoracolumbar burst fracture Posterior instrumented fusion	Diabetes, hypertension, heart diseases, prior brain attack	Risk factors and predictors for post-op recollapse
Kay et al. (35)	United States	Prospective	808	F = 406 M = 402 Mean age 58.1 ± 13.9	Degenerative lumbar disease (herniated disc, spondylolisthesis, spondylosis, stenosis) Laminectomy with fusion	Coronary artery disease, hypertension, previous myocardial infarction, congestive heart failure, diabetes mellitus, depression, and anxiety	Post-op ICU admission
Kaye et al. (36)	United States	Retrospective	50,495	Mean age: 66,75 ± 10,52 (with cardiac event) and 59,26 ± 13,58 (without cardiac event)	Different spine diseases Single-level lumbar spine fusion (arthrodesis with posterior, posterolateral, or anterior interbody technique, including minimal discectomy, laminectomy, and/or discectomy to prepare interspace)	Diabetes mellitus, congestive heart failure, renal failure, dialysis, peripheral vascular disease, chronic obstructive pulmonary disease, hypertension	Incidence and risk factors for adverse cardiac events
Khechen et al. (37)	United States	Retrospective	169	F = 69 females Mean age: 54.70 ± 11.36 M = 100 males Mean age 54.80 ± 11.32	Degenerative diseases Minimally invasive (MIS) TLIF	F: 2.1 ± 1.94 M: 2.10 ± 1.91	Post-op improvements in patient-reported outcomes after MIS TLIF
Kim et al. (38)	Korea	Retrospective	485	F = 329 M = 156 Mean age: 57.5	Degenerative spine diseases Partial hemilaminectomy, transpedicular screw fixation, and both posterior lumbar interbody fusion with cage and posterolateral fusion	Hypertension, cardiovascular diseases, renal diseases, pulmonary diseases, endocrine diseases, hepatic diseases F: 17.6% M: 14.7%	Complications after spinal surgery
Kothari et al. (39)	United States	Prospective	4,555	F = 2,686 M = 1,864	Spinal fusion deformities	Comorbidities: cardiac, pulmonary, and renal comorbidity, bleeding disorder, diabetes mellitus. Male ↑ cardiac events, bleeding disorder, diabetes mellitus	Post-op morbidity after spinal surgery
Lim et al. (40)	Southest Asia	Prospective	296	F = 202 Mean age: 52.2 ± 3.2 M = 94 Mean age: 56.1 ± 11.6	Grade 1 or 2 degenerative spondylolisthesis and nerve compression symptoms, radicular pain, paresthesia, or neurogenic claudication Single-level MIS TLIF	Diabetes, ischemic heart disease, osteoarthritis, asthma, depression, hypertension, hypercholesterolemia, and renal disease. Females ↑ prevalence of osteoarthritis	Functional, patient-reported outcome measures, health-related quality of life, satisfaction, and fulfillment of outcomes
Mai et al. (41)	Europe	Retrospective	10,770	F = 5,714 M = 5,056 Mean age: 62 ± 15	Degenerative diseases, complex deformity corrections Cervical interventions with anterior or posterior fusion (± instrumentation), thoracic/lumbar interventions with posterior decompression and/or spondylodesis with rigid stabilization either with posterior and/or interbody fusion (open or mini-open/MISS)	NR	LOS
Maior et al. (42)	Romania	Case series	44	F = 17 Mean age: 37.88 ± 13.56 M = 27 Mean age: 44.04 ± 14.28	Single-level traumatic burst fracture in the thoracolumbar transition area (Th12-L2)	NR	Health-related quality of life and disability after a thoracolumbar burst fracture
Malik et al. (43)	United States	Retrospective	23,615	F = 12,805 M = 10,810	Degenerative diseases one- to two-level posterior lumbar fusions	Diabetes mellitus, dyspnea, history of severe chronic obstructive pulmonary disease, congestive heart failure, hypertension, dialysis-dependent, bleeding disorders Females are more likely to be older and have dyspnea prior to surgery. Males were more likely to have diabetes and bleeding disorders	Early surgical outcomes after elective posterior lumbar fusions
Maragkos et al. (44)	United States	Retrospective	131	F = 78 M = 53 Mean age: 60	Degenerative spinal disease (discogenic, stenosis, spondylolisthesis), Posterolateral pedicular screw fixation with or without posterior interbody fusion	Diabetes, osteoporosis	Risk factors for development of adjacent segment disease
Marks et al. (45)	United States	Prospective	547	F = 449 Mean age: 14 ± 2 M = 98 Mean age: 16 ± 2	AIS	None	X-ray and perioperative surgical treatment outcomes of AIS
Nunley et al. (46)	United States	Prospective	389	NR	Cervical total disc replacement and cervical anterior discectomy and fusion	NR	Effects on outcomes, and risk factors for the development of heterotopic ossification after cervical total disc replacement
Ogihara et al. (47)	East Asia	Prospective	2,184	F = 682 M = 1,502 Mean age: 65.9	Degenerative diseases, spinal trauma, spinal tumor, or rheumatoid arthritis Posterior cervical instrumented fusion	Diabetes mellitus	Incidence of deep SSI development
Ogihara et al. (48)	East Asia	Prospective	2,913	F = 1,601 M = 1,312 Mean age: 65.9	Thoracic and/or lumbar degenerative diseases Posterior instrumented fusion	Diabetes mellitus	Risk factors for deep SSI
Park et al. (49)	East Asia	Prospective	16,927	F = 11,054 M = 5,873 Mean age: 62.35 ± 10.43	Degenerative lumbar disease (lumbar disc herniation, spondylolisthesis, stenosis) Single-level decompression and fusion	Diabetes, osteoporosis	Reoperation rates
Park et al. (50)	East Asia	Prospective	20,606	F = 13,122 M = 7,484 Mean age: 61.86 ± 10.97	Degenerative lumbar diseases single-level fusion (posterolateral fusion or posterior/transforaminal lumbar interbody fusion)	Diabetes, osteoporosis	Risk factors for repeat decompression and fusions
Parrish et al. (51)	United States	Retrospective	192	F = 77 Mean age: 53.2 ± 10.7 M = 115 Mean age: 50.6 ± 11.0	Degenerative spine diseases (herniated nucleus pulposus, degenerative disc disease, isthmic spondylolisthesis, spondylolisthesis, foraminal stenosis) Primary, one- or two-vertebral level MIS TLIF	Diabetes, diabetes mellitus	PROMIS-PF scores
Parrish et al. (52)	United States	Retrospective	75	F = 33 M = 42 Mean age: 49.9 ± 10.8	Degenerative spine diseases Primary, single-level MIS TLIF	Pre-op chronic health comorbidities: the most frequent were hypertension, arthritis, diabetes	Severity of PHQ-9 scores among patients with depressive symptoms
Poorman et al. (53)	United States	Retrospective	803,949	F = 410,818 M = 393,131 Mean age: 53.1	Different spine diseases Spinal fusion, decompression of the lumbar spine (laminectomy and discectomy with fusion)	Comorbidities associated with ↑ mortality: mild and severe liver disease, congestive heart failure	Rates and risk factors associated with mortality
Salzmann et al. (54)	United States	Retrospective	63	Fracture: *n* = 21 -F = 16 -M = 5 -Mean age: 66.4 Non-fracture: n = 42 -F = 32 -M = 10 -Mean age: 65.3	Sacral fractures after posterior instrumented fusion Posterior instrumented fusion	NR	Occurrence of post-op sacral fracture after spinal surgery
Samuel et al. (55)	United States	Retrospective	62,690	F = 26,818 M = 35,872	Degenerative spine diseases Single-level discectomy, or fusion	Overweight: 34.7% Obesity class 1: 24.9% Obesity class 2: 12.0% Obesity class 3: 7.7%	Rate of early failures after lumbar discectomy and associated risk factors
Schmitt et al. (56)	United States	Retrospective	172	F = 119 M = 53 Mean age: 61.8 ± 13.4	Long-segment fusion for adult spinal deformity correction	NR	Fusion rates using relatively low-dose rhBMP-2 for adult spinal deformity surgery
Shabat et al. (57)	Israel	Retrospective	367	F:177 M: 190 Mean age: ≥65	Degenerative spine disease (spondylolisthesis)	Obesity, depression, heart diseases, diabetes mellitus, osteoarthritis, peripheral vascular diseases	Patients’ satisfaction rates in lumbar spine surgery
Sharma et al. (58)	United States	Retrospective	191	F = 141 M = 50 Mean age: 64.3	Idiopathic, degenerative, or iatrogenic deformity Anterior, posterior, or circumferential lumbar and thoracolumbar fusion ≥3 levels	Obesity, diabetes II, hypertension, dyslipidemia	HRQoL
Smorgick et al. (59)	Israel	Retrospective	163	F = 128 Mean age: 15.15 M = 35 Mean age 16.17	AIS	None	Clinical and radiographic characteristics after AIS
Triebel et al. (11)	Europe	Prospective	4,772	F = 2,521 Mean age: 46 ± 11 M = 2,251 males Mean age: 46 ± 10	Degenerative disc disease and chronic low back pain Lumbar fusion	NR	Clinical outcomes
Ungureanu et al. (60)	Romania	Prospective	61	F = 31 Mean age: 48.67 ± 12.70 M = 30 Mean age: 50.93 ± 14.54	Degenerative pathology Posterior lumbar interbody fusion	NR	HRQoL and disability, and correlation between the two after spinal fusion for chronic low back pain
Wang et al. a (61)	East Asia	Retrospective	36	F = 20 M = 16 Mean age: 48.3	Cervical radiculopathy and myelopathy (revision surgery for symptomatic adjacent segment disease) Single-level ACDF	NR	Incidence and risk factors
Wang et al. b (62)	East Asia	Retrospective	153	F = 102 M = 51 Mean age: 50.11 ± 7.48	Cervical degenerative disc disease Continuous two- or three-level hybrid surgery (ACDF cervical disc replacement) for multilevel	NR	Impact of smoking on intermediate-term outcomes
Xu et al. (63)	China	Retrospective	162	F = 120 females Mean age: 14.79 M = 42 Mean age: 16.79	AIS Pedicle screw instrumentation and posterior fusion	None	Postoperative x-ray outcomes after AIS

M, male; F, female; NR, not reported; MIS, minimally invasive surgery; MISS, minimally invasive spine surgery; AIS, adolescent patients’ treated for idiopathic scoliosis; ACDF, anterior cervical discectomy and fusion; HRQoL, health-related quality of life; TLIF, transforaminal lumbar interbody fusion; LOS, length of stay; COPD, chronic obstructive pulmonary disease; SSI, surgical site infection; PROMIS-PF, Patient-Reported Outcome Measurement Information System-Physical Function.

**Table 2:** Clinical assessment and outcomes.

Ref.	FU	Assessment measures	Pre-op quantitative measures		Post-op quantitative measures		Complications	Specific sex/gender differences
Abousamra et al. (18)	NR	General health status, x-ray	NR		Mean magnitude of main thoracic curve = 60 ± 10 degrees. Mean measured 2D T5-T12 kyphosis = 22 ± 14 degrees and mean calculated 3D T5-T12 kyphosis = 6 ± 12 degrees.		NR	Male patients with severe thoracic lordosis (T5-T12 ≤ 0 degree) are the ↑ risk group for intra-op bleeding.
Adogwa et al. (19)	1 year 2 years	General health status	NA		NA		NR	Female sex, obesity, preoperative narcotic use, and LOS associated with prolonged opioid use after index surgery
Alomari et al. (20)	30 days	General health status	NA		NA		Peri-op blood transfusion, pulmonary embolism, deep vein thrombosis or thrombophlebitis, myocardial infarction, stroke with neurological deficit, unplanned intubation, pneumonia, deep or superficial SSI, sepsis, septic shock, death	Except for an ↑ incidence of UTI in female patients and myocardial infarction in males, no significant differences in morbidity and mortality
Alomari et al. (21)	30 days	General health status, laboratory values	NA		NA		-Peri-op blood transfusion: F = 141, M = 110 Pulmonary embolism: F= 13, M = 12 -DVT/thrombophlebitis: F = 22, M = 18 -Myocardial infarction: F = 7, M = 132 -Stroke with neurological deficit: F = 9, M = 4 -Unplanned intubation: F = 27, M = 26 -Pneumonia: F = 66, M = 57 -UTI: F = 344, M = 27 -Deep SSI: F: 13, M = 8 -Superficial SSI: F = 27, M = 10 -Sepsis: F = 26, M = 13 -Septic shock: F = 8, M = 12 -Death: F = 26, M = 26	Increased risk for UTI in females and myocardial infarction in males, no significant differences in morbidity and mortality between males and females
Ayhan et al. (22)	1 year	General health status, x-ray, self-reported HRQol measures (COMI, ODI, SF-36 MCS, SF-36 PCS, SRS-22)	MCCA = 40.85° T2–T12 kyphosis = 37.68 ODI = 40.41 SF-36 MCS = 41.48 SF-36 PCS = 35.94 SRS-22 Subtotal = 2.86		MCCA = 20.86° T2–T12 kyphosis = 45.05 ODI = 27.92 SF-36 MCS = 45.81 SF-36 PCS = 41.87 SRS-22 Subtotal = 3.53		NR	Gender does not have a significant effect on any of the HRQol scores
Basques et al. (23)	30 days	General health status	NA		NA		Pneumonia, reoperation, cardiac arrest, sepsis, unplanned intubation, ventilator for more than 48 h, death	The male gender is associated with a greater risk of any adverse event, also severe. Males with longer operative times compared to females (127 vs. 117 min)
Bumpass et al. (24)	2 years	General health status, x-ray, ODI, SF-36, SRS-22r	NR		NR		NR	Males have greater mean estimated blood loss. No gender differences in operative time, transfusion, final x-ray measurements, HRQoL, incidence of complications, also controlling for age, BMI, comorbidities, and levels fused
Buttermann et al. (25)	>10 years	VAS ODI x-ray	NR		NR		NR	ACDF outcomes not related to gender
Chan et al. (26)	3 months 12 months	General health status, ODI, EQ-5D, NRS-LP, NRS-BP	NR		NR		Readmission within 3 months of surgery: 9; Reoperation within 12 months: 13	Female sex is associated with most satisfaction (58.4% vs. 38.5%)
Chaichuangchok et al. (27)	6 months 12 months	JOA score	JOA score: 14.1 ± 2.7		JOA score: 15.9 ± 1.6		Transient hoarseness and dysphagia: *n* = 12	No significant difference of surgical outcome between genders; % of improvement of male patients ↑ than female
Christian et al. (28)	30 days	General health status	NA		NA		Wound infections, UTI, cases of pneumonia, myocardial infarctions, and thromboembolic events	Higher proportion of male smokers who underwent a PCDF compared to female smokers (9.93% vs. 5.98%)
Elsamadicy et al. (29)	At discharge	General health status	NA		NA		↑ peri-op transfusion in female than male patients; most prevalent complication in female patients is UTI, followed by pulmonary complications, hematomas, venous thromboembolism, and myocardial infarction. Most common complication in male patients is myocardial infarction, followed by pulmonary complications, cardiac complications, UTI, venous thromboembolism	Gender was not an independent predictor of discharge disposition, but was independently associated with increased LOS (female)
Gulbrandsen et al. (30)	6 weeks 3 months 6 months 1 year	VAS ODI x-ray	VAS: F = 6.54 vs. M= 6.14 ODI: F = 49.73 vs. M = 46.52		VAS: 6 weeks: F = 4.36 vs. M = 3.99 3 months, 6 months, and 1 year: no differences		NR	Females reported slightly more pain and worse function than males at the time of surgery, by 3 months no further gender differences in post-op pain or function existed
Helenius et al. (31)	M: mean 14.3 years F: mean 14.1 years	X-ray, scoliosis Research Society questionnaire, spinal mobility, and non-dynamometric trunk performance tests	Mean Cobb angle of the thoracic curve: 55° (range 42–83°) in the males and 56° (range 43–80°) in the females. The lumbar curves: 33° (range 10–59°) in males and 34° (range 21–64°) in females.		Final correction of the thoracic curves: 30% (range 19%–65%) in males and 33% (range 7%–71%) in females.		None	AIS provides similar short and long-term results in males and females
Hermansen et al. (32)	10 years	Clinically relevant improvement (C in neck-related pain intensity (≥30-mm improvement on a VAS), CRI in neck-specific disability (≥20% improvement in Neck Disability Index—NDI), radiological factors, EQ-5D, EQoL, VAS	NR		Median self-efficacy scale score: M = 170.5, F = 141.5 Median CSQ score—diverting attention: M = 9.0, F = 15.0; catastrophizing: M = 0.0, F = 8.5; praying/hoping: M = 2.0, F = 9.0; increased behavioral activity: M = 14.0, F = 17.5		NR	↑ neck- and arm-related pain intensity in females than males, more disability, and worse psychosocial status. Male gender was a predictor of CRI in neck-specific disability
Heyer et al. (33)	30 days	General health status	NA		NA		UTI: F = 1.96%; M = 0.89% Transfusion: F = 12.74%; M = 8.39% Unplanned intubation: F = 0.41%; M = 0.60% Pneumonia: F = 0.71%; M = 0.92% Superficial SSI: F = 0.92%; M = 0.70%	Differences in complications are present between males and females. Females were at increased risk for superficial SSI, UTI, transfusions, and longer LOS; males were at increased risk of pneumonia and reintubation
Jang et al. (34)	1 year	VAS, x-ray, CT, MRI, ODI	Measured body Ht. = 18.6 mm Body Ht. loss = 35.3% Body wedge angle = 18.9° Sagittal Cobb angle = 18.2°		Measured body Ht. = 26.7 mm Body Ht. loss = 8.4% Body wedge angle = 6.9° Sagittal Cobb angle = 4.6° Mean VAS score lower back pain = 3.25 ODI = 14.8		27 cases of failure of the screw-bone interface, 4 instrument failure, 2 revision surgeries	Recollapse showed ↑ a proportion of males
Kay et al. (35)	1 year	General health status, ODI%	ODI% = 48.2 ± 15.0		NR		Estimated blood loss = 12.2%; cardiac = 29.3%; respiratory = 19.5%; neurologic complications = 31.7%; other = 7.3%	Female gender, history of coronary artery disease, myocardial infarction, chronic heart failure, age, ASA grade, estimated blood loss, and LOS associated with ICU admission
Kaye et al. (36)	30 days	General health status	NA		NA		Myocardial infarction, sepsis, death	Factors associated with an increased cardiac risk after spinal fusion: age, male gender, insulin-dependent diabetes, ASA score >3, hematocrit, and smoking
Khechen et al. (37)	6 weeks 12 weeks 6 months	ODI VAS back and leg pain	ODI: F = 43.77 ± 16.38; M = 36.22 ± 15.13 VAS back: F = 6.62 ± 2.29; M = 5.90 ± 2.58 VAS leg: F = 6.20 ± 2.73; M = 5.27 ± 2.94		ODI: −6 weeks: F = −10.62 ± 17.63; M = −7.05 ± 19.15 −12 weeks: F = −18.49 ± 17.22; M = −13.53 ± 17.29 −6 months: F = −23.60 ± 17.76; M = −21.00 ± 15.98 VAS back: −6 weeks: F = −3.21 ± 2.94; M = −2.57 ± 3.15 −12 weeks: F = −3.63 ± 2.81; M = −2.92 ± 2.83 −6 months: F = −3.67 ± 3.24; M = −3.34 ± 3.16 VAS leg: −6 weeks: F = −3.48 ± 3.07; M = −3.01 ± 3.36 −12 weeks: F = −3.98 ± 2.94; M = −3.39 ± 3.12 −6 months: F = −4.40 ± 3.31; M = −3.32 ± 3.04		Intraoperative: none Inpatients: F = 2; M = 2 (altered mental status, urinary tract infection, intubation)	Gender is not associated with surgical or clinical outcomes; it is not a predictor of outcomes following MIS TLIF
Kim et al. (38)	>12 months	MacNab's criteria	NR		NR		None	Peri-op complications not significantly associated with gender
Kothari et al. (39)	30 days	General health status	NA		NA		Univariate analysis: F associated with ↑ intra-or post-op red blood cell transfusion, UTI, and LOS >5 days; M associated with ↑ rate of pulmonary and cardiac complications. Multivariate analysis: F predictor of any complication, intra- or post-op red blood cell transfusion, UTI, and LOS >5 days	Females are associated with ↑overall morbidity, i.e. for UTI, transfusion, and LOS > 5 days. Male associated with ↑incidence of pulmonary and cardiac complications
Lim et al. (40)	2 years 5 years	ODI, SF-36	ODI: M = 41.5 ± 18.0, F = 49.5 ± 17.3 SF-36 PCS: M = 35.6 ± 10.6, F = 31.9 ± 10.3 SF-36 MCS: M = 49.2 ± 11.7, F = 44.9 ± 12.3		NR		Revision surgery: F = 9, M = 3 Inadvertent durotomy: F = 4, M = 0. Vertebral endplate perforation: F = 1	Females are significantly younger than males at the time of surgery. At 2-year and 5-year follow-ups, no significant differences in ODI, SF-36, and pain scores between males and females
Mai et al. (41)	NR	General health status, COMI	NR		NR		General medical complications and surgical complications	↑ LOS in females than males. LOS ↑ with age in females compared to males. Female gender, age, and BMI associated with a longer LOS
Maior et al. (42)	1 year	SF-36v2 ODI	NR		NR		1 wound infection (F), 1 reinsertion of a wrongly inserted screw (M)	Gender-related differences favoring men after surgical interventions for spinal fractures. Male patients scored ↑ in each item of the SF-36v2. Male patients had ↓ ODI score
Malik et al. (43)	30 days	General health status	NA		NA		Deep surgical site infection, superficial surgical site infection, and sepsis/septic shock. The 3 most common reoperations in men: incision and drainage hematomas/seromas/fluid collections; incision and drainage, open, of deep abscess of posterior spine; reinsertion of spinal fixation device: in female: incision and drainage, open, of deep abscess of posterior spine; incision and drainage of complex wounds; and exploration of spinal fusion	Female gender is an independent risk factor significantly associated with a LOS longer than 3 days, occurrence of any complication within 30 days, wound complications, UTI, 30-day, 30-day readmissions, and nonhome discharge. The only adverse outcome associated with male was renal complication
Maragkos et al. (44)	NR	General health status, x-ray	NR		NR		Reoperation = 33	Decompression of segments outside the fusion construct associated with ↑ adjacent segment disease rates, as well as female gender
Marks et al. (45)	2 years	x-ray, pulmonary function data, SRS questionnaire	Curve magnitude: 55°±11° males and 54°±11° females, with flexibility of the primary curve ↓in males. % Predicted of FEV: 85%±18% in males versus 81%±16% in females; % of predicted FVC was 92%±20% in males versus 86%±16% in females. SRS scores are similar in males and females		% correction and ratio of % correction of the primary curve to preoperative flexibility: males 57% ± 17% and 1.58, females 60% ± 18% and 1.46. ↓ correction of the primary curve at 2 years FU minimal and comparable for males and females (2° ± 6° vs. 3° ± 7°, respectively). % Predicted FEV: 4% ± 12% in males and 5% ± 13% in females; decrease in % predicted FVC: 7% ± 13% in males and 6% ± 12% in females. SRS scores are similar in males and females		F: 5% perioperative complications, 2% major postoperative complication, 5% minor postoperative complications M: 8% perioperative complications, 6% major postoperative complications, 2% minor postoperative complications	X-ray and perioperative surgical treatment outcomes of AIS comparable between genders
Nunley et al. (46)	7 years	NDI VAS neck/arm pain SF-12	NR		NR		NR	Males were ∼3 times more likely to develop clinically relevant heterotopic ossification than females
Ogihara et al. (47)	1 year	General health status	NA		NA		Deep SSI	Risk factors for deep SSI: occipitocervical surgery and male gender
Ogihara et al. (48)	1 year	General health status	NA		NA		SSI	Independent risk factors: male gender, ASA score of ≥3, operation including thoracic spine
Park et al. (49)	4.5 years	General health status	NA		NA		Reoperation: >in patients with spinal stenosis than those with lumbar disc herniation	Male gender was a risk factor for reoperation
Park et al. (50)	90 days to 4 years	General health status	NA		NA		NR	Old age, and male gender, were risk factors
Parrish et al. (51)	6 weeks 3 months 6 months 1 year	General health status PROMIS-PF scores	NR		NR		NR	No significantly different among gender in the rate of achieving minimal clinically important difference for PROMIS-PF. Females experienced significantly more improvement in post-op PROMIS-PF scores than males at the 3-month time point
Parrish et al. (52)	6 weeks 12 weeks 6 months 1 year	PHQ-9 scores, SF-12, VR-12	38.7% mild (PHQ-9 score 5–9) 26.6% moderate (PHQ-9 score 10–14) 34.7% moderately severe (PHQ-9 score ≥15)		NR		NR	No significant differences between genders
Poorman et al. (53)	NR	General health status	NA		NA		Complications with the highest mortality rates: shock and pulmonary embolism	Increased mortality in males, black, ages 65–74, and age >75
Salzmann et al. (54)	≥6 months	CT	NR		NR		Post-op fracture	BMI and female gender are risk factors for post-op sacral fractures
Samuel et al. (55)	30 days	General health status	NR		NR		2.2% reoperation within 30 days: 1.2% spine-related reoperations, including 0.8% revision lumbar discectomy, 0.3% irrigation and debridement of infection, 0.1% lumbar fusion	Female gender is at increased risk of readmission for pain or neurological symptoms
Schmitt et al. (56)	2 years	X-ray	NR		NR		Constipation/ileus, hyponatremia, arrhythmia, UTI, anemia/coagulopathy, pneumonia, transient weakness/paresthesia superficial wound drainage/infection, urinary retention, hematoma, AKI, pseudarthrosis, instrumentation failure, proximal junctional kyphosis/adjacent segment disease, wound complication/infection with revision hemorrhage, cardiac arrest, stroke	Gender associated with fusion status, with 79.8% of females demonstrating fusion, compared to 60.4% of men
Shabat et al. (57)	1 year	VAS Barthel index (basic activities of daily status)	VAS: F 8.8 ± 1.86, M 8.36 ± 2.12 Barthel index: F 65.82 ± 11.08, M 68.28 ± 12.38		VAS: F 3.88 ± 2.56, M 3.39 ± 2.71 Barthel index: F 81.15 ± 12.24, M 84.14 ± 11.19		*n* = 1 death secondary to myocardial infarction; urinary retention, exacerbation of CHF and/or COPD, and unstable angina more frequently in males; urinary tract infection and postoperative delirium more frequently in females; *n* = 1 depression and CVA in females. Rate of complications similar among males (32%) and females (28%). More than one complication found in 30% of females and 20% of males.	Gender differences influence the satisfaction rate of lumbar spinal stenosis surgery, with females less satisficed than males
Sharma et al. (58)	1 year	HRQoL	NR		Improvement in EQ-5D scores in 98 patients (51%)		NR	Gender interacted with obesity: obese males with at higher odds of improvement when compared to nonobese males; among females, obesity did not affect the odds of improvement
Smorgick et al. (59)	None	VAS x-ray	NR		NR		None	Presence of a trend toward more flexible major thoracic curves in females but no significant difference between females and males
Triebel et al. (11)	1 year 2 years	General health status, Patient-Reported Outcome Measures, VAS for leg and back pain, ODI, HRQoL parameter EQ-5D	NR		NR		NR	Females with a ↑ rate of improvement from baseline to follow-up in leg pain, back pain, HRQoL, and disability. Females with better chances of clinical improvement than men for leg pain, back pain, and ODI, but improved at a slower pace in leg pain, back pain, and disability. No gender differences in HRQoL and return to work at 1-year and 2-year post-op
Ungureanu et al. (60)	1 year	SF-36v2 ODI	Female PCS: 27.08 ± 6.96 PF: 27.97 ± 8.46 RP: 27.02 ± 6.66 BP: 28.00 ± 4.86 GH: 40.53 ± 6.37 MCS: 46.54 ± 15.26 VT: 42.06 ± 10.23 SF: 39.23 ± 12.99 RE: 40.00 ± 15.14 MH: 40.66 ± 11.23 ODI: 55.91 ± 17.6 Pain intensity: 3.29 ± 1.13 Personal care: 2.00 ± 1.26 Lifting: 3.00 ± 1.44 Walking: 2.68 ± 1.28 Sitting: 3.35 ± 1.11 Standing: 3.39 ± 1.09 Sleeping: 1.97 ± 1.38 Sex life: 2.22 ± 1.83 Social life: 2.61 ± 1.65 Traveling: 3.13 ± 1.43	Male 9.20 ± 7.05 28.32 ± 7.87 29.39 ± 9.55 29.54 ± 6.62 47.78 ± 10.45 48.52 ± 14.79 42.99 ± 10.86 38.29 ± 13.96 42.47 ± 15.02 43.89 ± 12.62 56.19 ± 17.25 3.03 ± 1.00 2.37 ± 1.25 3.33 ± 1.40 2.93 ± 1.36 2.83 ± 1.60 3.27 ± 1.20 2.00 ± 1.36 2.64 ± 1.85 2.73 ± 1.64 2.97 ± 1.56	Female PCS:43.02 ± 9.76 PF: 44.95 ± 9.24 RP: 39.92 ± 10.03 BP: 43.78 ± 9.25 GH: 46.19 ± 10.78 MCS: 49.69 ± 9.83 VT: 51.93 ± 7.96 SF: 49.04 ± 8.92 RE: 46.06 ± 12.24 MH: 46.31 ± 9.16 ODI: 21.58 ± 15.35 Pain intensity: 1.1 ± 0.90 Personal care: 0.61 ± 0.80 Lifting: 2.13 ± 1.41 Walking: 1.00 ± 1.13 Sitting: 1.42 ± 1.31 Standing: 1.32 ± 1.38 Sleeping: 0.45 ± 0.85 Sex life: 0.83 ± 1.50 Social life: 0.90 ± 1.37 Traveling: 0.81 ± 0.95	Male 46.05 ± 10.03 46.63 ± 9.20 40.39 ± 11.20 49.39 ± 9.80 53.74 ± 9.83 53.32 ± 7.90 54.88 ± 8.88 50.66 ± 8.04 49.9 ± 10.44 50.17 ± 9.69 13.43 ± 10.33 0.97 ± 0.76 0.33 ± 0.71 1.83 ± 1.60 0.43 ± 0.63 0.80 ± 1.16 0.93 ± 1.11 0.30 ± 0.65 0.36 ± 0.99 0.23 ± 0.63 0.50 ± 0.94	NR	Male improved more than females in all domains of disability at the postoperative evaluation. HRQoL improved similarly in both genders. ODI score showed a strong or moderate correlation with six of the domains of the SF-36 in males, but with only three domains in females.	
Wang et al. (61)	5 years	General health status, x-ray	NR		NR	Loosening of the implant, collapse of the fusion intervertebral space, hematoma, deep infection		No gender differences
Wang et al. (62)	2 years	General health status, JOA scale, NDI, VAS for neck and arm pain, x-ray, CT, and MRI	NR	NR	NR		Male gender and current smoking status are significantly associated with a 1-year fusion rate. Significant differences in the early fusion process and the 1-year fusion rate across the three smoking status groups in females
Xu et al. (63)	3 months	X-ray	Sized main curves: F = 51.53°vs. M = 52.45°		Sized main curves: F = 16.83 vs. M = 20.8°		None	Comparable surgical benefits between females and males

NA, not applicable; CRI, Clinically relevant improvement; NDI, Neck Disability Index; ASA, American Society of Anesthesiologists physical status; COMI, Core Outcome Measures Index; PCS, physical component summary; PF, physical functioning; RP, role-physical; BP, bodily pain; GH, general health; MCS, mental component summary; VT, vitality; SF, social functioning; RE, role-emotional; MH, mental health; LOS, length of stay; UTI, urinary tract infection; HRQol, health-related quality of life; ODI, Oswestry Disability Index; SF-36, Short Form Health Survey 36; BMI, body mass index; VAS, visual analogue scale; ACDF, anterior cervical discectomy and fusion; NRS-LP, numeric rating scale for leg pain; NRS-BP, numeric rating scale for low back pain; JOA, Japanese Orthopedic Association; PCDF, posterior cervical decompression and fusion; ICU, intensive care unit; TLIF, transforaminal lumbar interbody fusion; SRS, Scoliosis Research Society; PROMIS-PF, Patient-Reported Outcome Measurement Information System-Physical Function; PHQ-9, Patient Health Questionnaire-9; COPD, chronic obstructive pulmonary disease; SRS-22, Scoliosis Research Society-22 questionnaire; MCCA, major coronal Cobb angle; EQ-5D, quality of life (QoL) parameter EQ5D; EQoL, Environmental Quality of Life; CSQ, Cognitive Style Questionnaire; FEV, forced expiratory volume; FVC, forced vital capacity; VR-12, Veterans RAND-12; SF-12, Short Form-12; AKI, acute kidney injury; CHF, chronic heart failure; CVA, Cerebrovasular accident.

Examining sex/gender differences was the primary objective in 25 (52.1%) studies while the remaining evaluated the influence of several predictors and risk factors (age, smoking history, number, and location of fusion segments, plate-to-disc distances, excessive disc space distraction, kyphotic malalignment), also including sex/gender as a secondary aim.

Comorbidities were assessed in 63.8% of the studies (*n* = 30). In two of these studies, no comorbidities were present. In the remaining 28 studies, different comorbidities were present that include diabetes (in 50% of included studies), cardiac diseases (25%) and/or hypertension (17.8%), blending disorders (14.2%), pulmonary diseases (10.7%), obesity and overweight (10.7%), osteoporosis (7.1%), dyslipidemia (7.1%), renal diseases (7.1%), osteoarthritis (7.1%), depression (7.1%), and others less common comorbidity, i.e., liver diseases, brain attack, vascular diseases, asthma, and anxiety. In 12/27 studies where comorbidities were present and described gender-related differences in comorbidities distribution were also reported. The major comorbidity reported in females was obesity while in males were diabetes, cardiovascular morbidities (prevalently atrial fibrillation and hypertension), and bleeding disorders.

Qualitative and quantitative measures used for patient assessment were detailed in Table 1. The Visual Analogue Scale (VAS) score for back or leg pain was used, alone or in association with other measurements, by 21.2% of the studies, the Oswestry Disability Index (ODI) by 25.5% of studies, x-ray, to evaluate pre-and postoperative sized main curves, sagittal Cobb angle, kyphosis, by 27.6% and specific patient-reported outcomes measures and standardized measure of health-related quality of life (HRQoL) by 42.5% of studies. Other less common measures used include the numeric rating scale (NRS) scores for low back pain (NRS-BP), leg pain (NRS-LP), Japanese Orthopedic Association (JOA) scale, magnetic resonance imaging (MRI), computed tomography (CT), and laboratory values. Except for one study that evaluated sex/gender differences at discharge, all other studies performed clinical assessments from 30-day of follow-up up to 10 years.

### Types of spine surgery and pathological conditions

Patients were diagnosed with different spine diseases, mainly degenerative pathological conditions such as disc herniation, stenosis, spondylolysis, radiculopathy, spondylolisthesis, myelopathy (55.3%), spinal deformities as adult scoliosis or kyphosis (12.7%), adolescent idiopathic scoliosis (10.6%), degenerative and deformity conditions together (14.8%), and traumatic pathologies such as fractures (6.3%). In detail, 235,140 patients (26 studies) were treated for degenerative diseases using anterior or posterior approaches (Table 1). Of these, 17 studies were retrospective and analyzed 188,278 patients, 8 were prospective and included 46,862 patients, and 1 study was an RCT with 72 patients. A total of five studies (four retrospective and one prospective) analyzed 1,769 adolescent patients' treated for idiopathic scoliosis (AIS) using a posterior approach. Concerning adult deformities, a total of 4,714 patients were evaluated in two prospective studies and 5,752 patients in four retrospective studies, using anterior or posterior approaches. Studies that analyzed degenerative and deformity conditions together (six retrospective studies and one prospective) evaluated 910,835 patients. Finally, 315 patients (three studies) were treated for traumatic sacral or thoracolumbar fractures after posterior instrumented fusion. Of these, two studies were retrospective with 271 patients, and one study was a case series with 44 patients.

Procedures associated with spinal fusion surgery included minor, major, and complex surgeries, such as posterior lumbar interbody fusion (PLIF), hemilaminectomy, decompression, anterior cervical discectomy and fusion (ACDF), transforaminal lumbar interbody fusion (TLIF), discectomy, anterior lumbar interbody fusion (ALIF) and laminectomy (Table 1). Because the types of spine fusion surgery were not standardized across the 47 studies, it was difficult to quantify the prevalence of any single type of procedure among the studies. However, most interventions were implemented for lumbar spine procedures, through techniques such as PLIF and TLIF, followed by cervical procedures such as ACDF.

### Sex/gender differences in outcomes after spinal fusion surgery

In this review, 76.6% (*n* = 36) of studies reported sex/gender-related differences in postoperative clinical outcomes and/or complications, while the remaining studies described comparable results between males and females (Table 2). Of these 36 studies where sex/gender differences were present, *n* = 21 (58.3%) were on degenerative diseases, prevalently of the lumbar spine, 13.9% were on spine deformities (1 on AIS and 4 on adult deformities), 8.4% on spine fractures, and 19.4% considered degenerative conditions and deformities together (Table 1).

### Degenerative spine diseases

More than half of studies on degenerative spine diseases on the lumbar spine reported worse female experiences after spinal fusion surgery (63.8%). Kay et al. showed that the female gender was associated with an increased risk of postoperative ICU admission in patients undergoing lumbar spine surgery (35). Furthermore, Maragkos et al. also reported that female patients were 2.55 times more likely than male ones to develop adjacent segment disease requiring reoperation (44). In detail, within 30 days of follow-up female gender resulted in an independent risk factor significantly associated with a LOS longer than 3 days, occurrence of complications, including wound complications, urinary tract infection (UTI), and nonhome discharge; while the adverse outcomes associated with males were renal complication and myocardial infarction (20, 43). At the same follow-up female gender also resulted in an increased risk of readmission for pain or neurological symptoms (55). At a longer follow-up, 1 year, it was analyzed the influence that gender plays on HRQoL, disability, and the correlation between these two in patients undergoing spinal fusion for chronic low back pain (60). Results showed that male patients had higher disability scores at the preoperative evaluation but improved more than females in all domains of disability postoperatively. HRQoL improved similarly in both genders. The ODI score showed a strong or moderate correlation with six of the domains of the Short Form Health Survey 36 (SF-36) in males, but with only three domains in females (60). At the same follow-up, it was also shown that gender differences influence the satisfaction rate of lumbar spinal stenosis surgery in elderly patients (≥65 years) with female patients that had less satisfactory results than male ones (57). After 2 years of follow-up, it was shown that the female gender was also associated with prolonged opioid use (19). Several studies reported different results. Parrish et al. reported that females experienced significantly more improvement in postoperative Patient-Reported Outcome Measurement Information System-Physical Function (PROMIS-PF) score than males at the 3-month time point (51). Chan et al. reported that at 1 year of follow-up, the female gender resulted also associated with more satisfaction than males (58.4% vs. 38.5%) (26). These results were further confirmed by Triebel et al. where it was shown that females had a high rate of improvement from baseline to follow-up (2 years) in leg pain, back pain, HRQoL, and disability, in comparison to males, but it was also reported that females improve more slowly in leg pain, back pain, and disability (11). At longer follow-up (up to 4.5 years), Park et al. showed that the male gender was a risk factor for reoperation (49, 50).

In cervical degenerative diseases at 1 year of follow-up a higher percentage of improvement was detected in male patients than in females (27). This result was also confirmed at 10 years of follow-up, where it was demonstrated a higher neck- and arm-related pain intensity, more disability, and worse psychosocial status in females than in males (32). Specifically, the male gender was a predictor of clinically relevant improvement in neck-specific disability (32). Differently, Nunley et al. (46) showed that males were ∼3 times more likely to develop heterotopic ossification than females as well as they were at greater risk of any adverse event (23). Alomari et al. (21) described an increased risk for UTI in females and myocardial infarction in males. Two studies also evaluated the correlation between gender, spinal fusion surgery, and smoking. A higher proportion of male smokers who underwent posterior cervical decompression and fusion (PCDF) compared to female smokers (9.93% vs. 5.98%) was detected (28). In addition, a significant difference in the early fusion process among current smokers, former smokers, and nonsmokers was detected for male patients, but no significant differences were founded at 1-year of follow-up. For female patients, statistical differences were found in both the early fusion process and the 1-year fusion rate (62).

The only study on sex/gender differences considering thoracic spine surgery was by Ogihara et al. using a multivariate analysis that showed that male gender was significantly correlated with a higher incidence of deep SSI, although it was not a significant factor in univariate analysis (48).

### Spine deformities

Of the 36 studies where sex/gender differences were evaluated, *n* = 4 were on adult spinal deformities. Two studies reported that female sex/gender was associated with increased postoperative morbidity compared to males; after 30 days from spinal surgery, it was observed an increased postoperative transfusion rate and complications, in particular, UTI and LOS longer than 5 days, in female patients (29, 39). Conversely, one study reported that male patients were at a greater risk for bleeding compared to females (24). Differently, from the above-mentioned studies, Schmitt et al. evaluated at 2 years of follow-up the fusion rates using relatively low-dose rhBMP-2 for the treatment of adult spinal deformity surgery, showing that gender was associated with fusion status, with 79.8% of females with a good spinal fusion compared to 60.4% of males (56).

Only one study on AIS detected gender-related differences, showing that male patients, with severe thoracic lordosis, were at a higher risk of bleeding than females (18).

### Degenerative conditions and deformities

Seven studies reported sex/gender-related differences considering patients treated for degenerative conditions and deformities together. In detail, at 30 days of follow-up, Heyer et al. (33) reported postoperative differences in complications between males and females. Females were at increased risk for superficial SSI, UTI, transfusions, and longer LOS while males were at increased risk of pneumonia and reintubation (33, 41). At the same experimental time, it was also shown that the male gender was associated with an increased risk of cardiac events (36). At a longer experimental time, 1-year follow-up, it was shown that the male gender represented a risk factor for deep SSI (47).

In a study on the mortality rates after spinal fusion surgery and on factors associated with its occurrence over a 10-year follow-up, it was shown that the male gender was a risk factor significantly associated with increased mortality rates (53). In addition to complications and risk factors related to gender, Gulbrandsen et al. (30) indicated that females reported slightly more pain and worse function than males at the time of surgery. Finally, Sharma et al. also showed that in spine surgeries gender interacted with obesity at 1 year of follow-up: obese males with at higher odds of improvement when compared to nonobese males; among females, obesity did not affect the odds of improvement (58).

### Spine fractures

Of the 35 studies where sex/gender differences were present, *n* = 3 were on spine fractures after spinal fusion surgery. One study was on sacral fractures (54), and two studies were on thoracolumbar burst fractures (34, 42). Salzmann et al. observed, at a 6-month follow-up, that female gender (76.2%), advanced age (mean, 66.4 years), and obesity were risk factors for post-op sacral fractures (54). Differently, Jang et al. showed that the male gender was a risk factor for recollapse of thoracolumbar burst fractures (34). At 1-year follow-up, Maior et al. reported that male patients have better outcomes than females after thoracolumbar burst fractures; an increased score in each item of the SF-36 in male than female patients was also observed (42).

## Discussion

From an epidemiological perspective, it would be critical to understand how sex/gender differences can affect clinical outcomes after spinal fusion surgery. This aspect becomes more critical from a surgical point of view since the identification of specific sex/gender differences would offer personalized approaches for patients undergoing spinal fusion procedures. Thus, the focus of this review was to analyze clinical studies aimed at evaluating sex/gender differences following spinal fusion surgery.

Our review showed that most of the analyzed studies (76.6%) report sex/gender-related differences in postoperative clinical status, outcomes, and/or complications, while the remaining described equivalent results. Furthermore, in 38.3% of the studies where comorbidities were described gender-related differences in their distribution were reported. In the female sex, obesity was the most prevalent comorbidity while in the male sex the prevalently dominant comorbidities were diabetes, cardiovascular morbidities (prevalently atrial fibrillation and hypertension), and bleeding disorders. These differences are of critical importance and could represent potential risk factors related to gender differences (both pre-op and post-op) to be analyzed in future studies, in which it will be mandatory to analyze a larger homogeneous cohort of patients, considering specific and individual spine diseases and surgical approaches. In fact, it is important to highlight that there is significant heterogeneity across the analyzed studies in terms of pathological spine diseases (degenerative, deformity, fracture), type of the spinal level treated (cervical, thoracic, lumbar, sacral), number of levels treated, and surgical approaches. In addition, it is important to underline that none of the analyzed studies evaluated specific physiological changes related to postmenopausal status in female patients submitted to spinal fusion procedures. It is known that the direct negative effects of estrogen-deficiency on bone as well as the indirect effects of altered immune status in postmenopausal women contribute to low bone mass and bone microarchitecture destruction. These bone alterations may represent a negative prognostic factor for the success of spinal fusion surgery.

In this review, most of the studies where sex/gender-related differences were present were on the patient treated for lumbar degenerative diseases (disc degeneration, disc herniation, and spondylolisthesis). Of those more than half reported a worse postoperative outcome in terms of pain, disability, HRQoL, and complications in female patients, while the remaining reported worse outcomes in terms of HRQoL and satisfaction in male patients. In studies examining pain, disability, and HRQoL differences between females and males, the complexity of evaluating these parameters which include many clinical signs and subjective outcomes must be considered. Undoubtedly, females treated for lumbar degenerative disease reported the worst postoperative scenario, but the perception and measurements of pain and disability have been debated and investigated: some studies concluded that females had lower perception thresholds and pain thresholds than males (64). This worst postoperative state in female patients is probably the reason for the prolonged postoperative opioid consumption associated with this gender (19). However, epidemiological studies have indicated that analgesic use may vary between males and females, suggesting that opioid use was higher in the female gender in general adult populations (65, 66). This systematic review found that females were also at increased risk of complications (UTI, readmission for pain or neurological symptoms, adjacent segment disease), and consequent longer LOS, following spinal fusion surgery for lumbar degenerative disease (20, 43, 44, 55). These postoperative complications may also have a link to the long-term opioid use detected in female patients. While for degenerative lumbar spine diseases a potential link between gender and clinical outcomes resulted more evident, for degenerative diseases of the cervical and thoracic spine this link is not so clear. This is probably due to the heterogenicity of the analyzed studies but also to the small number of studies found in this review. However, at 10-year to 13-year of follow-up, a prospective randomized study indicated that predictive factors of good outcome after ACDF included initial high neck-related pain intensity, nonsmoking status at the time of surgery, and male gender (32). On the contrary, other clinical studies indicated that males were more likely to develop clinically relevant heterotopic ossification than females as well as a greater risk of any adverse event, also severe (23). Similar contradictory results were also found after spinal deformity surgery, when degenerative diseases and deformities were considered and analyzed together and following spine fractures (sacral fractures and thoracolumbar burst fractures). Also, in these cases, the limited number of studies and the heterogeneity between studies remained critical factors that limit the interpretation of the results. For adult spinal deformities, a gender difference was founded by using relatively low-dose rhBMP-2 with the female gender demonstrating a better spinal fusion compared to of male one (56). In this study, there were substantial differences between the patient's series and those in the comparison groups. When degenerative diseases and deformities were considered and analyzed together it was also shown that gender interacted with obesity at 1 year of follow-up; obese males showed a higher odd of improvement when compared to nonobese males while among females, obesity did not affect the odds of improvement (58). Nevertheless, it should be noted that numerous studies present in the literature did not investigate and analyze spinal fusion outcomes dichotomizing by gender, thus it is difficult to establish a real conclusion and draw definitive results. As such, a true systematic review was not feasible. Furthermore, some limitations of this review warrant discussion. Most eligible studies in this review were retrospective and only one RCT on 47 analyzed studies was found and included. Additionally, many included studies had a small sample size and may be underpowered to identify significant clinical responses. Numerous studies, accounting for most patients, were prevalently published by groups in the United States; cultural discrepancies in patient-reported clinical assessment scores may differ geographically. The clinical assessment tools used to determine the postoperative outcomes are, prevalently, subjective patient-reported measures. Results were also difficult to interpret due to specific differences in spine diseases in the retrieved studies; for example, insufficient heterogenic data were retrieved for cervical and sacral degenerative diseases, spinal deformities, and traumatic spinal fractures. Furthermore, also searching for “spinal fusion” and “sex” or “gender” on the www.clinicaltrialg.gov website (accessed on 22 May 2022) no clinical trials had, as a primary or secondary outcome, the analysis of sex/gender differences.

## Conclusions

The review highlighted those female patients treated for lumbar degenerative spine diseases probably require more clinical awareness during postoperative care. The understanding of how sex/gender differences can really affect clinical outcomes after spinal fusion surgeries have the potential to enhance clinical decision-making and care practices and may be crucially important in the context of providing patient personalized care, a critical field of contemporary medical practice.

## Data Availability

The original contributions presented in the study are included in the article/Supplementary Material, further inquiries can be directed to the corresponding author.
