# Supplementary material for: Sex and gender determinants following spinal fusion surgery: A systematic review of clinical data
Source: Front Surg. 2022 Oct 17;9:983931. doi: 10.3389/fsurg.2022.983931 (PMC9618873; doi:10.3389/fsurg.2022.983931)
Supplement: Supplementary file 1 [file Table1.docx]

**SUPPLEMENTARY MATERIALS**

**Table S1: Combination of free-vocabulary and/or Medical Subject Headings (MeSH) terms for the identification of studies in PubMed, Scopus and Web of Science Core Collection**

| *PubMed:* (("spinal fusion"[MeSH Terms] OR ("spinal"[All Fields] AND "fusion"[All Fields]) OR "spinal fusion"[All Fields] OR ("spinal fusion"[MeSH Terms] OR ("spinal"[All Fields] AND "fusion"[All Fields]) OR "spinal fusion"[All Fields] OR ("spinal"[All Fields] AND "arthrodesis"[All Fields]) OR "spinal arthrodesis"[All Fields]) OR (("spine"[MeSH Terms] OR "spine"[All Fields] OR "vertebral"[All Fields] OR "vertebrals"[All Fields]) AND ("fusions"[All Fields] OR "gene fusion"[MeSH Terms] OR ("gene"[All Fields] AND "fusion"[All Fields]) OR "gene fusion"[All Fields] OR "fusion"[All Fields])) OR (("spine"[MeSH Terms] OR "spine"[All Fields] OR "vertebral"[All Fields] OR "vertebrals"[All Fields]) AND ("arthrodesed"[All Fields] OR "arthrodesing"[All Fields] OR "arthrodesis"[MeSH Terms] OR "arthrodesis"[All Fields] OR "arthrodese"[All Fields] OR "arthrodeses"[All Fields]))) AND ("sex characteristics"[MeSH Terms] OR ("sex"[All Fields] AND "characteristics"[All Fields]) OR "sex characteristics"[All Fields] OR ("gender"[All Fields] AND "differences"[All Fields]) OR "gender differences"[All Fields] OR ("sex characteristics"[MeSH Terms] OR ("sex"[All Fields] AND "characteristics"[All Fields]) OR "sex characteristics"[All Fields] OR ("sex"[All Fields] AND "differences"[All Fields]) OR "sex differences"[All Fields]) OR "gender-specific"[All Fields] OR "sex-specific"[All Fields])) AND ((y_10[Filter]) AND (english[Filter])) |
| --- |
| *Scopus:* ( spinal AND fusion OR spinal AND arthrodesis OR vertebral AND fusion OR vertebral AND arthrodesis ) AND ( gender AND differences OR sex AND differences OR gender-specific OR sex-specific ) AND ( LIMIT-TO ( PUBYEAR , 2022 ) OR LIMIT-TO ( PUBYEAR , 2021 ) OR LIMIT-TO ( PUBYEAR , 2020 ) OR LIMIT-TO ( PUBYEAR , 2019 ) OR LIMIT-TO ( PUBYEAR , 2018 ) OR LIMIT-TO ( PUBYEAR , 2017 ) OR LIMIT-TO ( PUBYEAR , 2016 ) OR LIMIT-TO ( PUBYEAR , 2015 ) OR LIMIT-TO ( PUBYEAR , 2014 ) OR LIMIT-TO ( PUBYEAR , 2013 ) OR LIMIT-TO ( PUBYEAR , 2012 ) ) AND ( LIMIT-TO ( DOCTYPE , "ar" ) ) AND ( LIMIT-TO ( LANGUAGE , "English" ) ) |
| *Web of Science Core Collection:* (TS = spinal fusion OR TS = spinal arthrodesis OR TS = vertebral fusion OR TS = vertebral arthrodesis) AND (TS = gender differences OR TS = sex differences OR TS = gender-specific OR TS = sex-specific)—with Publication Year from 2012 to 2022, English |

**Table S2. Risks of bias assessments in a randomized trial.**

| **Reference** | **Bias Domain** | | | | | |
| --- | --- | --- | --- | --- | --- | --- |
|  | **Bias arising from the randomization process** | **Bias due to deviations from intended interventions** | **Bias due to missing outcome data** | **Bias in measurement of the outcome** | **Bias in selection of the reported result** | **Overall bias** |
| Hermansen et al. 2013 |  |  |  |  |  |  |

**Key:**

Low risk of bias

High risk of bias

Some concerns

**Table S3. Risks of bias assessments in a non-randomized study.**

| **Reference** | **Bias Domain** | | | | | | | |
| --- | --- | --- | --- | --- | --- | --- | --- | --- |
|  | **Bias due to confounding** | **Bias in selection of participants into the study** | **Bias in classification of interventions** | **Bias due to deviations from intended interventions** | **Bias due to missing data** | **Bias in measurement of the outcome** | **Bias in selection of the reported result** | **Overall bias** |
| Gulbrandsen et al. 2021 |  |  |  |  |  |  |  |  |
| Kim et al. 2013 |  |  |  |  |  |  |  |  |
| Buttermann et al. 2018 |  |  |  |  |  |  |  |  |
| Salzmann et al. 2019 |  |  |  |  |  |  |  |  |
| Smorgick et al. 2019 |  |  |  |  |  |  |  |  |
| Xu et al. 2015 |  |  |  |  |  |  |  |  |
| Khechen et al. 2019 |  |  |  |  |  |  |  |  |
| Samuel et al. 2021 |  |  |  |  |  |  |  |  |
| Alomari et al. 2021a |  |  |  |  |  |  |  |  |
| Chaichuangchok et al. 2017 |  |  |  |  |  |  |  |  |
| Ungureanu et al. 2018 |  |  |  |  |  |  |  |  |
| Nunley et al. 2018 |  |  |  |  |  |  |  |  |
| Kothari et al. 2016 |  |  |  |  |  |  |  |  |
| Elsamadicy et al. 2020 |  |  |  |  |  |  |  |  |
| Maior et al. 2018 |  |  |  |  |  |  |  |  |
| Alomari et al. 2021b |  |  |  |  |  |  |  |  |
| Malik et al. 2018 |  |  |  |  |  |  |  |  |
| Schmitt et al. 2016 |  |  |  |  |  |  |  |  |
| Bumpass et al. 2017 |  |  |  |  |  |  |  |  |
| Mai et al. 2020 |  |  |  |  |  |  |  |  |
| Heyer et al. 2019 |  |  |  |  |  |  |  |  |
| Sharma et al. 2019 |  |  |  |  |  |  |  |  |
| Kay et al. 2016 |  |  |  |  |  |  |  |  |
| Maragkos et al. 2020 |  |  |  |  |  |  |  |  |
| Poorman et al. 2018 |  |  |  |  |  |  |  |  |
| Park et al. 2019a |  |  |  |  |  |  |  |  |
| Park et al. 2019b |  |  |  |  |  |  |  |  |
| Jang et al. 2018 |  |  |  |  |  |  |  |  |
| Kaye et al. 2018 |  |  |  |  |  |  |  |  |
| Ogihara et al. 2021a |  |  |  |  |  |  |  |  |
| Ogihara et al. 2021b |  |  |  |  |  |  |  |  |
| Basques et al. 2018 |  |  |  |  |  |  |  |  |
| Adogwa et al. 2019 |  |  |  |  |  |  |  |  |
| Christian et al. 2020 |  |  |  |  |  |  |  |  |
| Wang et al. 2017 |  |  |  |  |  |  |  |  |
| Wang et al. 2021 |  |  |  |  |  |  |  |  |
| Ayhan et al. 2018 |  |  |  |  |  |  |  |  |
| Parrish et al. 2020 |  |  |  |  |  |  |  |  |
| Parrish et al. 2021 |  |  |  |  |  |  |  |  |
| Abousamra et al. 2019 |  |  |  |  |  |  |  |  |
| Triebel et al. 2017 |  |  |  |  |  |  |  |  |
| Lim et al. 2020 |  |  |  |  |  |  |  |  |
| Chan et al. 2018 |  |  |  |  |  |  |  |  |
| Marks et al. 2007 |  |  |  |  |  |  |  |  |
| Shabat et al 2005 |  |  |  |  |  |  |  |  |
| Helenius et al. 2005 |  |  |  |  |  |  |  |  |

**Key:**

Low risk of bias

Moderate risk of bias

Critical risk of bias
